# Temporal changes in transcriptome profile provide insights of White Spot Syndrome Virus infection in *Litopenaeus vannamei*

**DOI:** 10.1038/s41598-019-49836-0

**Published:** 2019-09-18

**Authors:** Luca Peruzza, M. S. Shekhar, K. Vinaya Kumar, A. Swathi, K. Karthic, Chris Hauton, K. K. Vijayan

**Affiliations:** 1 School of Ocean and Earth Science, University of Southampton, Hampshire, SO14 3ZH United Kingdom; 2 Genetics and Biotechnology Unit, Central Institute of Brackishwater Aquaculture, 75 Santhome High Road, R.A. Puram, Chennai, 600004 Tamil Nadu India

**Keywords:** Viral infection, Transcriptomics, Animal physiology

## Abstract

Shrimp aquaculture is severely affected by WSSV. Despite an increasing effort to understand host/virus interaction by characterizing changes in gene expression (GE) following WSSV infection, the majority of published studies have focussed on a single time-point, providing limited insight on the development of host-pathogen interaction over the infection cycle. Using RNA-seq, we contrasted GE in gills of *Litopenaeus vannamei* at 1.5, 18 and 56 hours-post-infection (hpi), between WSSV-challenged and control shrimps. Time course analysis revealed 5097 differentially expressed genes: 63 DEGs were viral genes and their expression in WSSV group either peaked at 18 hpi (and decreased at 56 hpi) or increased linearly up to 56 hpi, suggesting a different role played by these genes during the course of infection. The remaining DEGs showed that WSSV altered the expression of metabolic, immune, apoptotic and cytoskeletal genes and was able to inhibit NF-κB and JAK/STAT pathways. Interestingly, GE changes were not consistent through the course of infection but were dynamic with time, suggesting the complexity of host-pathogen interaction. These data offer novel insights into the cellular functions that are affected during the course of infection and ultimately provide a valuable resource towards our understanding of the host-pathogen dynamics and its variation with time.

## Introduction

Over the last decade, marine shrimp aquaculture has experienced one of the fastest development rates of all industrial sectors^1,2^ and the whiteleg shrimp *Litopenaeus vannamei* is, at present, the dominant crustacean species farmed worldwide^2,3^ with an annual production in 2016 exceeding 4 × 10^6^ tons (http://www.fao.org/fishery/culturedspecies/Penaeus_vannamei/en#tcNA008C) worth over >U.S. $11 billion^3^. In spite of this economic expansion, in the last 30 years this sector has been plagued by the emergence of viral diseases (e.g. White Spot Disease (WSD), Taura syndrome or Yellowhead disease^1,4^), with important global economic and social repercussions^5^. In fact, the incidence of disease in aquaculture is a serious threat for the economy of countries that greatly rely on aquaculture^4^ and is a threat to the availability of food security for the human population^5^.

In 1991 WSD was first reported in shrimp farms in China and Taipei^4^ and since then it has spread globally (cumulative losses exceeded $10bn in the period 1993–2006^4,6^). WSD is caused by the White Spot Syndrome Virus (WSSV), a double strand DNA (dsDNA) virus^7^, that can be transmitted horizontally^8^ (by water-borne contact or ingestion). WSSV has a broad range of host organisms, with data identifying susceptibility in 47 species, and has numerous aquatic organisms that are reported as mechanical vectors^4^. In the majority of penaeid shrimps, the later stages of the infection cause a reddish discolouration of the body or the appearance of white spots on the carapace^6^ with cumulative mortality that can reach 100% within 10 days following the onset of disease^7^.

As stated by Rao *et al*.^9^, in order to develop a pro-active approach to suppress the disease, a better understanding of host-pathogen interaction is needed. Indeed, over the last years, we have seen an increasing effort in trying to characterize host-pathogen interaction from a physiological^10–13^ and molecular^9,14–23^ point of view. As an example, an increasing amount of evidence is currently reporting major metabolic changes during WSSV infection (i.e. an increased cellular metabolism coupled with an increased aerobic glycolysis, known as the Warburg effect^24,25^) and alterations in haematological parameters (e.g. total haemocyte count, hemocyanin, clotting of haemolymph, phenoloxidase^11,13,26^). In addition, molecular and transcriptomic studies have reported alterations in Gene Expression (GE) of haemolymph coagulation^15,16,20,23^, apoptosis^23,27^, ubiquitination^28^, heat shock proteins^9,20^, RNA-mediated silencing^9^, growth-related genes^20^ and immune genes^29^ (e.g. *C-type lectins, Penaeidins*) and activation/inhibition of pathways related to immunity^27,30,31^ (e.g. NF-κB, JAK/STAT and Wnt pathways). All these results underline major changes in the physiology of infected hosts and suggest the complexity of host-pathogen interaction. Very recently, new studies have also shed light into the genome of *L. vannamei*^32^ and into single nucleotide polymorphisms (SNPs) associated with tolerance traits^33^ that will prove useful in explaining this host-pathogen interaction.

However, the interaction between host and pathogen is dynamic and varies during the course of infection: for example, Li *et al*.^25^ reported that WSSV is able to trigger a metabolic boost to support its replication in the initial stage of infection, while, in the late stage of infection WSSV is able to subvert again host metabolic processes to facilitate viral assembly and release. Given this temporal evolution of the infection, it can be argued also that GE may change during the course of infection. Therefore, in order to fully understand the mechanism of infection it is essential to characterize these dynamic changes in GE. Hitherto, the vast majority of available transcriptomic studies focussed on comparing GE between infected and control animals at a single time point^9,14–19^, therefore providing no insight on how GE in host and pathogen is changing with time. In order to improve our knowledge on the dynamic interaction between host and pathogen with time, we used an RNA sequencing (RNA-seq) approach to contrast GE changes in gills of *L. vannamei* at different stages of infection, namely 1.5, 18 and 56 hours-post-infection (hpi), between WSSV-challenged shrimps and control shrimps. We implied a Time Course analysis approach^34^ to identify genes with significant temporal expression changes and significant differences between experimental groups. We identified 5097 differentially expressed genes (DEGs), grouped in 9 temporal patterns of expression. 63 DEGs were viral genes with two different temporal expression profiles and the remaining DEGs, grouped into different pathways, revealed how multiple cellular processes were dynamic during the course of infection. These data offer novel insights into the cellular functions that are affected during the course of infection and ultimately provide a valuable resource towards our understanding of the dynamics between host and pathogen and its variation with time.

## Results

### Viral loads

**Figure 1:**
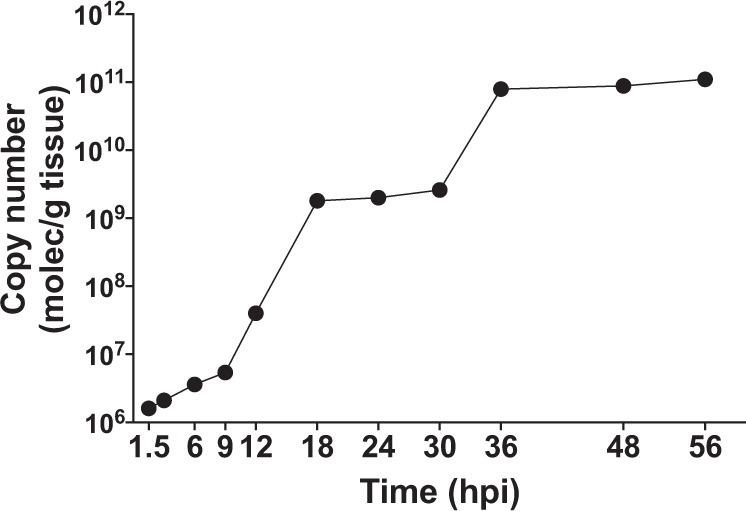
Viral load. The number of copies of WSSV detected at different time points after injection of WSSV in *L. vannamei* (dots represent the average copy number from n = 3 independent biological replicates per time point).

Viral loads in gill tissue increased with time from 1.6E + 06 at 1.5 hpi to 1.1E + 11 at 56 hpi (Fig. 1). Interestingly viral load increased near exponentially from 1.5 to 18 hpi (reaching 1.8E + 09 copy numbers), was constant until 30 hpi and then increased again after 30 hpi, reaching a second plateaux at 36 hpi (reaching 7.9E + 10 copy numbers).

### Transcriptome assembly

Illumina RNA sequencing was conducted on gill tissue extracted from *L. vannamei* (WSSV infected, “WSSV”, and control, “Ctrl”) at 1.5 hpi (“WSSV 1.5 h”, “Ctrl 1.5 h”), 18 hpi (“WSSV 18 h”, “Ctrl 18 h”) and 56 hpi (“WSSV 56 h”, “Ctrl 56 h”) from 3 different biological samples per treatment per time point (total n = 18). These three time points were chosen because they represented three distinct phases of the infection (Fig. 1): an initial stage at 1.5 hpi where the virus was actively replicating (in terms of copy numbers) in the host; an intermediate phase at 18 hpi where WSSV copy number reached a first plateaux; a late phase at 56 hpi where there WSSV copy number reached a second plateaux. After 56 hpi some deceased animals were observed (data not shown), confirming the late stage of the infection.

After cleaning the raw reads with Trimmomatic, a total of 199,795,880 reads were used to perform *de novo* assembly. Assembly generated 1,351,294 contigs with an N50 value of 359 (Table 1). The completeness and integrity of the assembled transcriptome were assessed with BUSCO, which revealed that 97.9% of the benchmarking orthologous genes were present in the initial assembly (Table 1 and Suppl. Table 1). The initial assembly was subsequently reduced to 26,054 contigs (N50 = 3409) after normalisation using RUVs package (to account for multiple sequencing runs and remove unwanted variation) and removal of contigs poorly covered (i.e. CPM < 5, Table 1). The number of annotated transcripts was 10391 (39.88%, Table 1). Raw sequence data associated with this project have been deposited at NCBI with bioproject accession number PRJNA524934 and SRA accession numbers from SRR8654034 to SRR8654051.

**Table 1:** Summary of assembly statistics for the transcripts obtained from *L. vannamei* infected and non-infected with White Spot Syndrome Virus.

Statistics	Initial Assembly	CPM Filter (>5 cpm)
Assembled transcripts	1351294	26054
Average Length	387	1890
Transcript N50	359	3409
BUSCO Assembly	97.90% Complete	—
	1.4% Fragmented	
	0.7% Missing	
# Blasted transcripts	—	11227 (43.09%)
# Annotated transcripts	—	10391 (39.88%)

A principal component analysis (PCA) was performed to compare replicates across samples. PCA showed that “WSSV samples” clustered tightly and were separated from “Ctrl samples” (Suppl. Fig. 1), indicating that infection with WSSV altered the overall GE profile at all time points.

### Time course (TC) expression analysis

**Figure 2:**
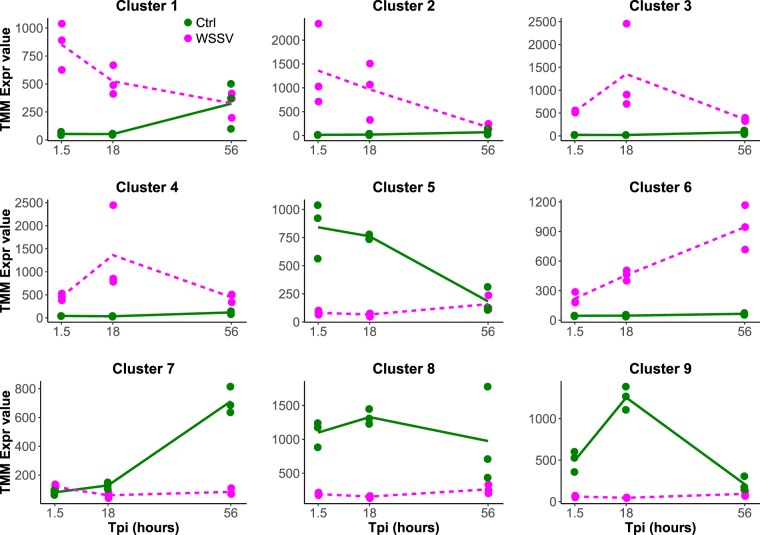
Time Course Analysis. Graphs represent the 9 clusters of expression in which the 5097 differentially expressed genes (in *L. vannamei* infected or non-infected) were divided based on their expression profile. In each graph, dots represent the median expression level for each biological replicate in each time point (n = 3 different biological replicates for each treatment (e.g. Ctrl and WSSV infected) for each time point (e.g. 1.5 h, 18 h and 56 h)). For each treatment, the average expression levels in each time point are connected by a line. Green dots and lines = Ctrl while purple dots and lines = WSSV infected.

TC analysis allowed us to identify 5097 genes with significant temporal expression changes and differences between treatments (i.e. “Ctrl” and “WSSV”, Suppl. Tables 2 and 3). These genes were grouped in 9 different cluster of expressions (Fig. 2). Genes in clusters 1, 2, 3, 4 and 6 were up-regulated in WSSV group, while genes in clusters 5, 7, 8 and 9 were up-regulated in Ctrl group. Enrichment analysis on all up-regulated genes in WSSV group revealed an over-representation of categories involved in sugar uptake across membranes (i.e. “phosphoenolpyruvate-dependent sugar phosphotransferase system” and “protein-N(PI)-phosphohistidine-sugar phosphotransferase activity”), of “ATP-binding cassette (ABC) transporters” and “viral envelope” (Suppl. Table 4). Within the clusters up-regulated in WSSV group, temporal changes were observed: genes in cluster 1 and 2 had the highest expression level at 1.5 hpi, and then GE decreased up to 56 hpi. Genes in cluster 3 and 4 had a bell-shaped expression pattern, with a peak in expression at 18 hpi; finally, genes in cluster 6 increased linearly their expression over the course of infection.

### Differentially expressed viral genes

**Figure 3:**
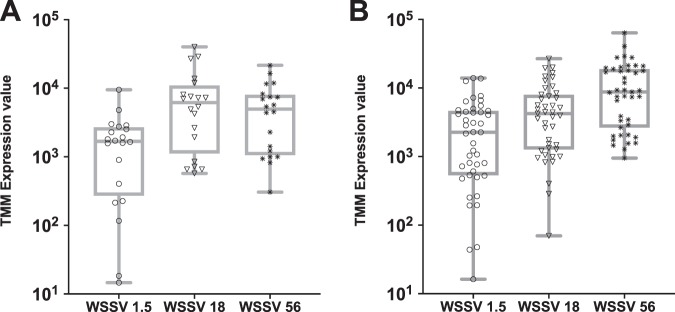
Differentially expressed viral genes from TC analysis. (**A**) Boxplots of differentially expressed viral genes (in *L. vannamei* infected or non-infected) with a bell-shaped profile of expression with time (e.g. between 1.5 h and 56 h). Each symbol represents the average expression level (as trimmed means of M values (TMM)) of a single viral gene. (**B**) Boxplots of differentially expressed viral genes with a linear expression profile with time (e.g. between 1.5 h and 56 h). Each symbol represents the average expression level (as trimmed means of M values (TMM)) of a single viral gene. A list of all DE viral contigs is found in Suppl. Table 5.

TC analysis revealed that 63 viral contigs were DE in WSSV group. Although the majority of viral contigs have unknown function, we detected some known genes, such as a *TATA-box binding protein TBP*, a *microtubule-binding protein MIP-T3* and several envelope, capsid and virion proteins (Suppl. Table 5). The temporal pattern of expression of these contigs could be divided in two groups: a first group with a bell-shaped pattern, with a peak in expression at 18 hpi (Fig. 3A); a second group with an expression level that increased linearly with time (Fig. 3B).

### Cellular processes and pathways affected during WSSV infection

**Figure 4:**
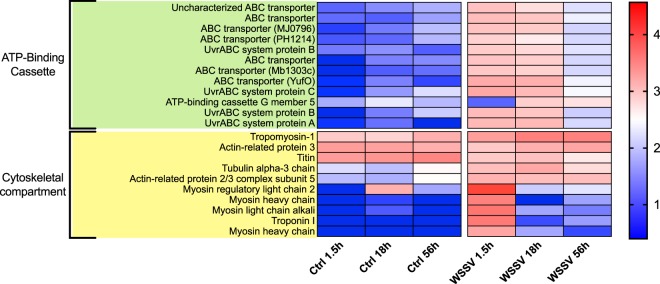
Heatmap of ABC transporter DE genes and cytoskeletal DE genes. Heatmap showing DE genes (in *L. vannamei* infected or non-infected) mapping to ABC transporter genes and cytoskeletal genes in control (“Ctrl”) and WSSV infected (“WSSV”) animals at different time points (e.g. 1.5 h, 18 h, 56 h). Each cell in the heatmap represents the average expression level from three independent biological replicate samples. Colour legend is on a log10 scale. Trinity contig names matching each gene can be found in Suppl. Table 6.

**Figure 5:**
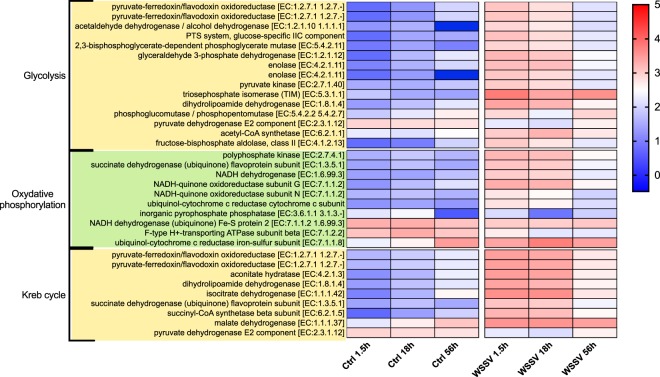
Heatmap of DE genes associated with metabolism. Heatmap showing DE genes (in *L. vannamei* infected or non-infected) mapping to three metabolic processes (e.g. Glycolysis, Oxidative phosphorylation and Kreb cycle) in control (“Ctrl”) and WSSV infected (“WSSV”) animals at different time points (e.g. 1.5 h, 18 h and 56 h). Each cell in the heatmap represents the average expression level from three independent biological replicate samples. Colour legend is on a log10 scale. Trinity contig names matching each gene can be found in Suppl. Table 6.

**Figure 6:**
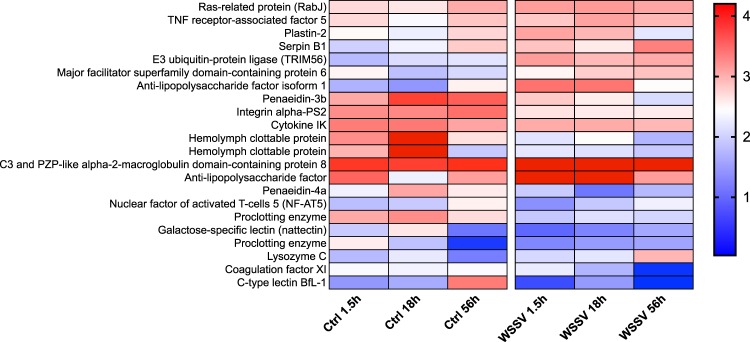
Heatmap of immune DE genes. Heatmap showing DE genes (in *L. vannamei* infected or non-infected) mapping to immune genes in control (“Ctrl”) and WSSV infected (“WSSV”) animals at different time points (e.g. 1.5 h, 18 h and 56 h). Each cell in the heatmap represents the average expression level from three independent biological replicate samples. Colour legend is on a log10 scale. Trinity contig names matching each gene can be found in Suppl. Table 6.

**Figure 7:**
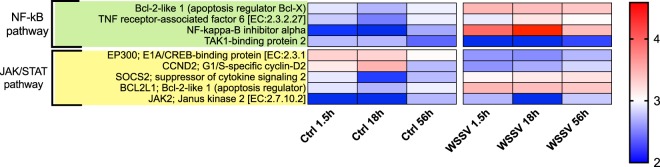
Heatmap of DE genes associated with immune pathways. Heatmap showing DE genes (in *L. vannamei* infected or non-infected) mapping to immune pathways (e.g. NF-kB pathway and JAK/STAT pathway) in control (“Ctrl”) and WSSV infected (“WSSV”) animals at different time points (e.g. 1.5 h, 18 h and 56 h). Each cell in the heatmap represents the average expression level from three independent biological replicate samples. Colour legend is on a log10 scale. Trinity contig names matching each gene can be found in Suppl. Table 6.

**Figure 8:**
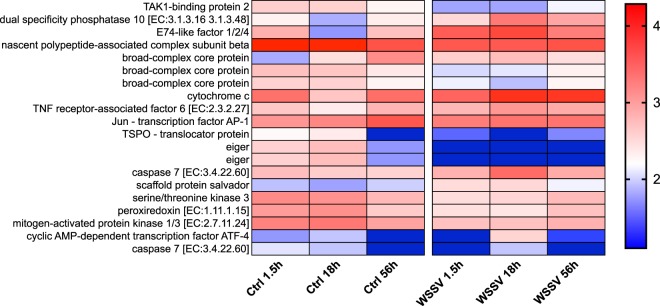
Heatmap of DE genes associated with apoptosis. Heatmap showing DE genes (in *L. vannamei* infected or non-infected) mapping to apoptotic processes in control (“Ctrl”) and WSSV infected (“WSSV”) animals at different time points (e.g. 1.5 h, 18 h and 56 h). Each cell in the heatmap represents the average expression level from three independent biological replicate samples. Colour legend is on a log10 scale. Trinity contig names matching each gene can be found in Suppl. Table 6.

KEGG analysis on genes identified from TC analysis allowed the identification of pathways that were altered during the process of infection (Suppl. Table 6). Overall, we found changes in GE with time between treatments (i.e. Ctrl and WSSV groups) and within WSSV treatment (i.e. between different time points, Figs 4–8 and Suppl. Figs 2, 3). We found a strong up-regulation of 11 ABC transporters in WSSV group at 1.5 and 18 hpi (Fig. 4), whereas the expression levels of the same genes decreased at 56 hpi in WSSV group. Interestingly, a similar pattern of expression was noticed for enzymes related to DNA duplication and maintenance (Suppl. Fig. 2) and for 30 metabolic genes mapping to glycolysis, Krebs cycle or oxidative phosphorylation (Fig. 5). We observed an up-regulation of several genes belonging to the cytoskeletal compartment in WSSV group: *Myosin heavy and light chains*, *Troponin-1* and *Tropomyosin-1, Tubulin* and *Actin-related protein 2/3* (Fig. 4); these genes were highly upregulated in WSSV group at 1.5 hpi and their expression decreased at 18 hpi in WSSV group. We identified an up-regulation of genes mapping to the clathrin-dependent endocytosis process in WSSV group at all time points (Suppl. Fig. 3) and we found a strong up-regulation of TAR1, Transcript Antisense to Ribosomal RNA, which is able to maintain oxidative phosphorylation capacity in mitochondria and can trigger production of reactive oxygen species, ROS (Suppl. Fig. 4). Several immune related genes were up-regulated in WSSV group compared to Ctrl group (e.g. *Serpin B1*, *Anti-lipopolysaccaride factor*, Fig. 6), although the majority of immune genes were down-regulated in WSSV group, including *Penaeidin-3b* and *-4a* and genes involved in the coagulation cascade (e.g. *Haemolymph clottable protein*, *proclotting enzyme*). The expression of two pathways (i.e. NF-κB and JAK/STAT) associated with immunity in shrimp was investigated and revealed that both pathways were inhibited (Fig. 7) due to the strong up-regulation of negative regulators for these pathways: *NF-κB inhibitor alpha* for NF-*κ*B pathway, and *Suppressor of cytokine signalling 2, SOCS2*, for JAK/STAT pathway. In addition, we investigated expression levels of genes related to apoptotic processes and we found that, at 1.5 and 18 hpi, there were more genes up-regulated in Ctrl than in WSSV group (the average expression level was double in Ctrl compared to WSSV groups, Fig. 8); inversely, at 56 hpi, the majority of apoptotic genes were up-regulated in WSSV group (e.g. *Dual specificity phosphatase 10*, *E74-like factor*, *Cytochrome c*, *TSPO*, *Scaffold protein Salvador*, *Peroxiredoxin*, *Ser/Thre Kinase 3*).

### Validation of bioinformatic analysis via qPCR

**Figure 9:**
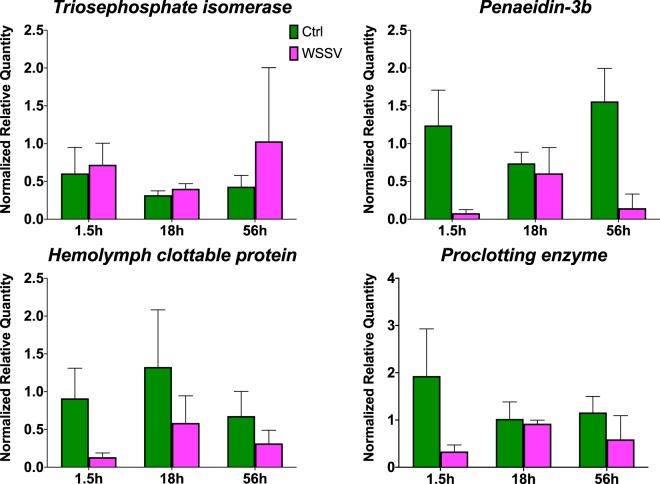
Confirmatory qPCRs of selected DE genes. Normalised Relative Quantities of selected genes from the DE genes using Elongation factor as housekeeping gene at each time point (e.g. 1.5 h, 18 h and 56 h). Data are shown as mean + SD of n = 3 independent biological replicates. Green columns represent Ctrl values, while purple columns represent WSSV infected values.

Quantitative PCR analysis on four DEGs confirmed the bioinformatic analysis (Fig. 9): the metabolic gene *Triosephosphate isomerase*, which was up-regulated in WSSV animals at RNA-seq, was found higher in WSSV animals after qPCR analysis; on the other hand, the immune genes *Penaeidin-3b, Hemolymph clottable protein* and *Proclotting enzyme* were down-regulated in WSSV infected animals at RNA-seq level and following qPCR analyses.

## Discussion

In the present study we investigated the relation between *L. vannamei* and WSSV during the course of infection by utilising a Time Course analysis, which allowed us to compare GE at three different time points, 1.5, 18 and 56 hpi, and revealed dynamic changes in GE during the course of infection. In the virus, we identified that expression of viral genes could be divided into two profiles of expression (i.e. bell-shaped and linear expression profiles, Fig. 3) and, within the host, we found up-regulation of ABC transporters and cytoskeletal genes, down-regulation of several immune genes, inhibition of NF-*κ*B and JAK/STAT pathways and changes in several metabolic and apoptotic genes. Interestingly, in WSSV group, the abovementioned changes were not consistent with time and in the majority of cases the expression level of one gene in the late stage of infection (i.e. 56 hpi) markedly differed from the expression level in the early-middle stage of infection (i.e. 1.5 and 18 hpi): for example, this difference was found in the majority of metabolic, apoptotic or ABC transporter genes. Overall, results underline that the process of infection is dynamic and that host-pathogen interaction can result in relevant GE changes during the course of infection.

### Viral gene expression

TC analysis identified in total 63 WSSV genes as differentially expressed between Ctrl and WSSV groups. Among them, we found up-regulation of TRINITY_DN588797_c0_g1_i2, blasting a viral *TATA-box binding protein, TBP*. As reported by van Hulten *et al*.^35^ this *TBP* may function similarly to eukaryotic TBPs, which bind to the TATA-box promoter region of many eukaryotic genes and start the transcription mediated by the RNA polymerase. Arguably this TBP would play an important role during infection, as major WSSV structural virion protein genes, including immediate-early protein 1 (IE1), possess a TATA-box sequence upstream their start codon^36,37^; hence this endogenous viral TBP could facilitate and initiate the transcription of viral genes. In addition we found up-regulation of TRINITY_DN570955_c1_g3_i1, blasting a viral *protein kinase*, and TRINITY_DN589043_c9_g8_i1, blasting a *Microtubule-binding protein MIP-T3*, capable of binding microtubular structures and inducing the reorganization of microtubular cytoskeletal network in the cells^38^. The cytoskeleton plays an important role during WSSV infection, as recently shown by Tang *et al*.^28^, which reported how actin, a component of the cytoskeletal microfilaments, is phosphorylated by a tyrosine kinase during WSSV infection. This phosphorylation causes a post-translational modification (PTM) that can affect actin’s activity, cellular location or interaction with other proteins^28^, and hence, together with the microtubular reorganization induced by *MIP-T3*, may facilitate viral hijacking of the cells.

Not all viral genes that were identified with TC analysis had the same expression profile with time (Fig. 3). Interestingly, while the majority of them linearly increased their expression levels during the course of infection, some had a bell-shaped profile, peaking at 18 hpi and then decreasing their expression at 56 hpi. It could be argued that genes peaking at 18 hpi may play an important role in the initial stages of infection (e.g. while hijacking cell machinery or inducing viral GE) and their expression decreases towards the later stages of infection when their function becomes less important. In agreement with this hypothesis, we observed that *TBP* (involved in inducing viral GE), *Vp9* (involved in viral genome replication^39^) and *MIP-T3* (able to induce microtubular reorganization) had a bell-shaped profile, while genes that linearly increased their expression levels were mostly structural viral genes, such as *VP24*, *VP26* or envelope proteins and their expression is increasing in later stages of infection when new viral particles are being produced, in agreement with the morphogenesis model from Escobedo-Bonilla *et al*.^7^.

### Host gene expression

To date many transcriptomic studies have reported changes in GE resulting from WSSV infection, but so far these studies were limited on a single time point^9,14–19,21^, providing no insight into dynamic changes in GE happening during the course of infection. The use of TC analysis allowed us to identify genes with significant differences between treatments and between different time points that were subsequently mapped to identify altered cellular processes or pathways during WSSV infection.

In order to successfully replicate, viral particles initially need to enter the cell of the host^7^. TC analysis revealed up-regulation of 12 ATP-binding cassette proteins in WSSV group at 1.5 and 18 hpi. These proteins can transport substrates across membranes by using ATP^40^ and, given the strong up-regulation detected in our data, it can be hypothesised that ABC transporters may be linked to the process of infection (either as a viral entry mechanism into the cell or as a host defensive mechanism). This hypothesis is supported by reports of ABC transporters being significantly changed upon WSSV infection in L. vannamei^23^, of ABC transporters being up-regulated in silkworm cells infected with *Bombyx mori* nucelopolyhedrovirus^41^ and of ABC transporters inhibiting HIV-1 infectivity in human T cells^42^. Interestingly, the expression levels of ABC transporters from Sagisaka *et al*.^41^ in silkworm cells increased up to the late stages of infection and was followed by an abrupt decrease^41^. A similar trend was found in our dataset, with the expression of ABC transporters decreasing by ~6 times from 18 hpi to 56 hpi in WSSV group (Fig. 4), further suggesting an involvement of ABC transporter as entry mechanism or host defensive mechanism in initial stages of infection. In addition, Du and Jin^19^ and Chen *et al*.^17^ found GE changes in ABC transporters after WSSV infection in red swamp crayfish gills and whiteleg shrimp hepatopancreas: interestingly, while in whiteleg shrimp hepatopancreas ABC transporters were down-regulated, in red swamp crayfish gills ABC were upregulated, suggesting that an important difference exists between these two tissues.

Another mechanism to enter host cell is via the endocytic route mostly by using clathrin, caveolae or pinocytotic processes^43,44^. In accordance with Huang *et al*.^44^ and Chen *et al*.^43^, we observed an up-regulation of genes mapping to the clathrin-dependent endocytosis process (i.e. *clathrin heavy chain*, *HSP70kDa*, *phospholipase D1/2*, *TNF receptor-associated factor 6*, Suppl. Fig. 3) suggesting that the virus is using clathrin mediated endocytosis to enter gill cells. After successfully entering into the cell, WSSV nucleocapsid is transported to the nucleus where viral genome is released and viral replication starts^7^. It is interesting to note that in our dataset we found a strong up-regulation of DNA-related enzymes (e.g. *DNA polymerase*, *DNA ligase*) in WSSV group at 1.5 and 18 hpi, while GE levels were, on average, decreased by ~10 times at 56 hpi. This observation supports the hypothesis that viral replication takes place mostly in the initial stages of infection and that newly synthetized viral particles are then subsequently assembled into the cell^7^, thus decreasing the need for enzymes like DNA polymerase and explaining the drop in their expression.

Cytoskeletal genes, such as *actin*, *myosin* or *tubulin* were all up-regulated in WSSV group at 1.5 and 18 hpi (Fig. 4). On average, the overall expression level for this group of genes was ~107 times higher in WSSV group at 1.5 hpi compared to Ctrl group at 1.5 hpi, while it was only ~1.5 times higher at 18 hpi. As previously mentioned, WSSV is able to phosphorylate actin via a tyrosine kinase to affect its activity^28^ and, by expressing viral *MIP-T3*, WSSV is able to bind cytoskeletal proteins and induce the reorganization of microtubular network^38^. In addition, we found the down-regulation in WSSV group of TRINITY_DN582016_c3_g1_i1, blasting a *Ras association domain-containing protein 8*, a protein essential in maintaining actin-cytoskeletal organization^45^, which may be actively targeted by WSSV. Hence, it can be argued that, in the initial stages of infection (i.e. from 1.5 hpi to 18 hpi), WSSV induces major changes to the cytoskeletal compartment (probably while hijacking cell machinery), and these modifications trigger a cellular response that up-regulates cytoskeletal genes. This hypothesis is in accordance with the up-regulation of *myosin light chain* in WSSV infected *Fenneropenaeus chinensis* reported by Shi *et al*.^20^ and with ESTs, mapping *actin*, *myosin light chain* and *troponin I*, over-expressed in WSSV infected *L. vannamei* and reported by Alcivar-Warren *et al*.^46^.

In total 30 metabolic enzymes were found up-regulated in WSSV group at 1.5, 18 and 56 hpi. It is widely appreciated that viral infections alter the metabolism, which commonly translates into changes of metabolite concentrations^12,47–49^ to support bioenergetic and biosynthetic demands of viral replication^25^. In this study, the upregulated genes code for proteins of the glycolysis and Krebs cycle and suggested a generalized metabolic boost, in agreement with Li *et al*.^25^, Chen *et al*.^43^ and Wang *et al*.^21^ who reported an enhancement in aerobic glycolysis (i.e. Warburg effect) and/or TCA cycle (i.e. Krebs cycle) during the replication stage of infection (i.e. within 24 hpi). It is interesting to note that our data also suggested an up-regulation of oxidative phosphorylation (in contrast to previous studies), further confirmed by the strong up-regulation of *TAR1* (Suppl. Fig. 4); in fact this gene is able to maintain mitochondrial oxidative phosphorylation capacity^50^ and may enhance ROS production as a defence mechanism against WSSV. In addition, our results indicated an important temporal change in GE of metabolic genes which were, on average, reduced by ~78% in WSSV group at 56 hpi (compared to 1.5 hpi). This drop in GE indicates a change in cellular metabolism and it is concordant with the hypothesis that, to facilitate viral assembly and release in the late stage of infection, WSSV is able to subvert host metabolic processes, as reported by Li *et al*.^25^.

In this study, many immune genes were altered in animals infected with WSSV (Fig. 6) in agreement with Ren *et al*.^22^, with the majority of them being down-regulated, including antimicrobial peptides (AMPs) such as *Penaeidin-3b* and *Penaeidin-4a* or lectins (*C-type lectin BfL-1)*. Several authors have argued that Penaeidins have a role against viruses (see^29,51^) and their downregulation may be the direct result of the action of the virus to avoid the immune system of the host. In support to this hypothesis we found that *Penaeidins* were consistently down-regulated across all time points in our data and in data from Zhang *et al*.^29^. Lectins are essential pattern-recognition proteins of the innate immune system of arthropods and, in a similar way to Penaeidins, Liu *et al*.^52^ and Ma *et al*.^53^ reported their down-regulation after WSSV infection. Interestingly, in the resistant species *M. rosenbergii* an opposite pattern in GE was observed for many AMPs; in fact, AMPs such as lectins, galectins and the anti-lipopolysaccharide factors were all up-regulated following WSSV infection^9,15^, suggesting important differences between the species.

In addition to AMPs, we found that several components of the haemolymph coagulation cascade (e.g. *Proclotting enzyme, Haemolymph clottable protein*) were down-regulated. The coagulation cascade is a component of the humoral immunity and several components of this physiological mechanisms were found down-regulated at all time points in WSSV group (Fig. 6). Arguably the down-regulation of clotting proteins may indicate that the virus is actively regulating the process of coagulation during infection, since evidence from GE studies^20,23,51^ and physiological studies^11^ report down-regulation of haemolymph clotting genes and absence of haemolymph coagulation in the vulnerable species *L. vannamei, Penaeus indicus* and *Fenneropenaeus chinensis*. It is interesting to note that, in the resistant prawn *Macrobrachium rosenbergii and Macrobrachium japonicus*, different components of the coagulation cascade were found to be up-regulated after WSSV challenge^9,15,16^ and in *M. rosenbergii* haemolymph was still able to clot after WSSV infection^13^. Hence, these differences in the coagulation system and in GE of AMPs coupled with the different susceptibility of the species should arguably require a detailed investigation as they may be important in determining WSSV vulnerability in *L. vannamei* and *P. indicus*.

Apoptosis is an important regulatory mechanism to prevent mutagenesis^54^ and to defend the body against virus infection^27^ and several authors reported changes in apoptosis following WSSV infection^25,55,56^. In our dataset we found 20 DE genes mapping to apoptotic processes. At 1.5 and 18 hpi the majority of them (~65%) were up-regulated in Ctrl group, while at 56 hpi ~75% were up-regulated in WSSV group (Fig. 8). This suggests an important shift during the course of infection as in the initial stage the virus may inhibit apoptosis to favour its replication, while in the late stage of infection the virus may trigger apoptosis to facilitate the spread of newly assembled viral particles, in agreement with Wongprasert *et al*.^56^ who showed that, in *Penaeus monodon* gills infected with WSSV, apoptotic cells start appearing only after 24 hpi. In fact, Shekhar and Ponniah^27^ and Graidist *et al*.^57^ suggested that “apoptosis is part of the pathophysiology that leads to shrimp death” since, in the late stage of infection, organelles (including mitochondria) disintegrate and nuclear and cellular membranes of infected cells are disrupted^7^ eventually leading to apoptosis.

In invertebrates several pathways have been associated with an immune response, including the JAK/STAT and the NF-*κ*B pathways^30,31^. In our dataset we found an inhibition of JAK/STAT pathway at all time points due to the up-regulation of its major inhibitor, *Suppressor of cytokine signalling SOCS2*, which was also found up-regulated by Du *et al*.^14^ in crayfish intestine. This gene is a regulator that exerts a negative feedback on STAT and Wang *et al*.^58^ demonstrated that shrimp knockdown for *SOCS2* exhibit increased WSSV resistance. In fact, the inhibition of this pathway may have a direct effect on the production of AMPs such as *Penaeidins*, as demonstrated by Sun *et al*.^59^. Hence, we can hypothesise that WSSV is able to inhibit the antiviral response that the JAK/STAT pathway would trigger by regulating *SOCS2* expression. Interestingly, despite the overall inhibition of JAK/STAT pathway, we found an up-regulation of *Janus kinase 2 JAK2*. This up-regulation can be explained by the fact that JAK is putatively involved in other pathways^30^ and may be responsible for the up-regulation of antiviral molecules (such as Serpin B1, Fig. 6) independently of STAT, in agreement with Song *et al*.^30^, suggesting that WSSV is not able to completely suppress host antiviral response in *L. vannamei*.

In a similar way we found that also NF-*κ*B pathway was inhibited due to the strong up-regulation of *NF-κB inhibitor (IκB)* in WSSV group at all time points. NF-*κ*B is an anti-apoptotic pathway and is also responsible for the production of AMPs^60^. Interestingly the expression of *IκB* in WSSV group peaked at 18 hpi and was decreased by 10 times at 56 hpi (Fig. 7), putatively suggesting that the inhibition of NF-*κ*B pathway is more important in the peak phase rather than in the late stage of the infection. Further, Wang *et al*.^31^ demonstrated how, for a successful infection, the kinases that regulate *IκB* levels (e.g. IKKß and IKKε) need to be expressed, because their presence can induce the promoters of many WSSV immediate-early genes, while their silencing results in resistance to WSSV. Interestingly, *IKKε* was present in our transcriptome and its GE was not different between Ctrl and WSSV groups, suggesting that WSSV could exploit *IKKε* to promote transcription of viral genes while suppressing NF-*κ*B pathway and consequently blocking the production of AMPs.

## Conclusions

In order to improve our knowledge on host-pathogen interaction we performed a Time Course analysis to find DE genes with statistical temporal differences among and within treatments. TC allowed us to identify differences in GE between three stages of the infection (1.5, 18 and 56 hpi) and, more importantly, showed that WSSV and host GE was dynamic during the course of infection. Hence, we hypothesize that, in the early stage of infection (1.5 hpi and 18 hpi) the virus entered the cells (via endocytosis and/or ABC transporters), where it triggered modifications to the cytoskeletal network, probably to facilitate hijacking cell machinery. Soon after infection (1.5 hpi) WSSV was able to boost cellular metabolism (i.e. Warburg effect) to support its replication (via expression of several DNA polymerases). Simultaneously WSSV interfered with the host immune system (i.e. down-regulating synthesis of AMPs and altering haemolymph clotting system) by inhibiting immune related pathways (i.e. JAK/STAT and NF-κB) and suppressed apoptosis. Once viral replication was completed (after 24 hpi) and virion particles needed to be assembled, WSSV was able to induce another shift in host metabolism, underlined by the decrease in expression (~78%) of metabolic genes at 56 hpi. Finally, once viral assembly was completed (~56 hpi), WSSV was able to induce apoptosis in infected cells to facilitate the spread of new viruses. Collectively, our results provide experimental evidence that different biological processes were altered at different times during the infection, highlighting the temporal complexity of host-pathogen interactions. We argue that in order to fully understand the mechanisms of WSSV infection and, ultimately, unravel the complexity of host-pathogen interaction, an approach that implies multiple observations at different stages of the infection is essential.

## Materials and Methods

### Experimental procedure

For each time point juvenile *L. vannamei* shrimps (n = 3) were injected with an intramuscular injection of WSSV (virus isolate: WSSV_CIBA_003, GenBank accession no. MH883319). Viral dose was 100 µl of 10^−1^ dilution of viral stock containing 5.3 × 10^7^ μl^−1^ of viral copies. Control animals (n = 3 per each time point) were injected with sterile Phosphate-buffered saline (PBS).

### Viral loads

To monitor the increase in viral load with time, gill tissues from WSSV-infected and control shrimps were dissected at eleven time points (i.e. 1.5 h, 3 h, 6 h, 9 h, 12 h, 18 h, 24 h, 30 h, 36 h, 48 h and 56 h post-WSSV infection stage). DNA was extracted from all samples using a QIAamp DNA Mini Kit (QIAGEN, Hilden, Germany) and tested for WSSV infection by nested PCR using WSSV gene specific primers^61^. Viral copy number was estimated by qPCR using TaqMan Universal Master Mix II (ABI technologies, USA).

### RNA extraction and library preparation

For each time point (i.e. 1.5, 18 and 56 hpi) gill samples were dissected from WSSV-infected and control juveniles *L. vannamei* (n = 3 per time point) and RNA was extracted. RNA sequencing libraries were prepared with Illumina-compatible NEBNext® Ultra™ Directional RNA Library Prep Kit (New England BioLabs, MA, USA) following manufacturer protocol and Illumina multiplex barcode adapters were used. The final PCR product (sequencing library) was purified with HighPrep beads, followed by library quality control check. The Illumina-compatible sequencing library was initially quantified by Qubit fluorometer (Thermo Fisher Scientific, MA, USA) and its fragment size distribution was analyzed on Agilent 2200 TapeStation (Agilent Technologies, Waldbronn, Germany).

### Transcriptome assembly, annotation and analysis

Raw reads from WSSV infected libraries (WSSV group, n = 9 total libraries) and control libraries (Ctrl group, n = 9 total libraries) were quality checked using FastQC/v0.11.3 (https://www.bioinformatics.babraham.ac.uk/projects/fastqc/) and low quality reads were removed using Trimmomatic^62^/v0.32 with default parameters. All 18 libraries were used for *de novo* transcriptome assembly using Trinity^63^/v2.4.0 with “—min_kmer_cov 2” and default parameters. Transcriptome redundancy was reduced using CD-HIT/v4.6.4^64^ with default parameters and the quality of the assembly was assessed using BUSCO/v3.0.2^65^ using the arthropoda_odb9 database. Reads were mapped to the transcriptome using Kallisto^66^/v0.43.1. The functions “betweenLaneNormalization” and “RUVs” from the R/v3.5.0^67^ package RUVSeq^68^/v1.14.0 were used to normalize the dataset and remove unwanted variation. The count matrix and the transcriptome were then filtered to retain contigs with CPM > 5 in at least 2 libraries, therefore removing contigs with low coverage which may derive from mis-assembly or contamination and contribute to background noise^69,70^. After filtering, Principal Component Analysis was then performed in R using the package RUVSeq.

The filtered transcriptome was annotated against UniProt^71^, NCBI nr and Pfam^72^ databases with BlastX/v2.6.0^73^ (e-value 1E-05 and default options) and InterPro Scan^74^/v5.29–68.0 (default options). Hits were imported in Blast2GO^75^/v5.2.5 and GO numbers were retrieved using “mapping” and “annotation” functions with default options.

### Time course expression analysis

The count matrix and transcriptome were imported in Blast2GO where the Time Course (TC) Analysis^34^ was performed with default parameters. This tool applies a two-step regression analysis to identify genes with significant temporal expression changes and significant differences between experimental groups. Statistical significance was identified at p-value < 0.05 with an R^2^-cutoff > 0.7.

All clusters containing genes up-regulated in WSSV group were merged in Blast2GO to perform Enrichment Analysis with Fisher’s Exact test with FDR p-value < 0.05.

All significant features from TC analysis were submitted to the KEGG^76^ Automatic Annotation Server (KAAS, http://www.genome.jp/tools/kaas/) to retrieve KEGG pathway maps for each contig using GHOSTZ with the single-directional best hit (SBH) method. The expression levels for each gene in each pathway was extracted from Blast2GO and used to plot the heatmaps in Prism/v8.0.2 (GraphPad Software, California USA, www.graphpad.com) after log_10_ transformation of the data.

### Validation of bioinformatic analysis via qPCR

The expression of some DEGs was further confirmed by means of quantitative PCR analysis. For each biological sample (n = 3 independent biological samples per treatment per time point, n = 18 biological samples in total) a volume containing 1.5 μg of total RNA was treated with Promega RQ1 RNase-free DNase (Promega Corporation, Hants, UK) according to the manufacturer’s protocol. Total RNA (0.68 μg) was reverse transcribed in a 20 μl reaction using Superscript III reverse transcriptase (Invitrogen, UK) and oligo(dT)20 primers.

All qPCR reactions were performed on a ABI StepOne Plus thermocycler (Life Technologies Corporation). qPCR reaction conditions were: Initial PCR heat activation at 95 °C for 10 min, followed by 40 cycles of 95 °C, 15 s; 60 °C, 1 min. The specificity of the PCR products was detected by melt curve analysis 95 °C, 15 s; 60 °C, 1 min and 95 °C, 15 s. Each reaction was run in duplicate (technical replicate). In addition NTC (no template control samples in which no DNA was added) were run. Primer-sets used are reported in Suppl. Table 7.

Elongation factor 1 was used as reference gene in all run. The expression level of each gene of interest was expressed as 2^−∆∆Ct^, in accordance with Rao *et al*.^77^, by using Elongation factor 1 as reference gene.

## Supplementary information

Supplementary Figures

Suppl Tab 1

Suppl Tab 2

Suppl Tab 3

Suppl Tab 4

Suppl Tab 5

Suppl Tab 6

Suppl Tab 7

## Data Availability

Raw sequence data associated with this project have been deposited at NCBI with bioproject accession number PRJNA524934 and SRA accession numbers from SRR8654034 to SRR8654051.
