# Supplementary material for: Temporal changes in transcriptome profile provide insights of White Spot Syndrome Virus infection in Litopenaeus vannamei
Source: Sci Rep. 2019 Sep 18;9:13509. doi: 10.1038/s41598-019-49836-0 (PMC6751192; doi:10.1038/s41598-019-49836-0)
Supplement: Supplementary file 1 — Supplementary Figures [file 41598_2019_49836_MOESM1_ESM.docx]

**Temporal changes in transcriptome profile provide insights of White Spot Syndrome Virus infection in *Litopenaeus vannamei***

Luca Peruzza*^1^, M.S. Shekhar^2^, K. Vinaya Kumar^2^, A. Swathi^2^, K. Karthic^2^, Chris Hauton^1^ and K.K. Vijayan^2^

^1^ School of Ocean and Earth Science, University of Southampton, Hampshire, SO14 3ZH, United Kingdom

^2^ Genetics and Biotechnology Unit, Central Institute of Brackishwater Aquaculture, 75, Santhome High Road, R.A Puram, Chennai

Supplementary Material

Supplementary Figure 1: Principal Component Analysis (PCA). PCA showing the differences between the independent biological samples analysed from infected (WSSV) and non-infected (Ctrl) *L. vannamei* at different time points (e.g. 1.5h, 18h and 56h). Each symbol represents an independent biological replicate sample from different treatments at different time points.

Supplementary Figure 2: Heatmap of DE genes involved in DNA maintenance/replication. Heatmap showing DE genes involved in DNA maintenance/replication in control (“Ctrl”) and WSSV infected (“WSSV”) *L. vannamei* at different time points (e.g. 1.5h, 18h, 56h). Each cell in the heatmap represents the average expression level from three independent biological replicate samples. Colour legend is on a log10 scale. Trinity contig names matching each gene can be found in Suppl. Table 6.

Supplementary Figure 3: Heatmap of DE genes associated with endocytosis. Heatmap showing DE genes involved in endocytosis processes in control (“Ctrl”) and WSSV infected (“WSSV”) *L. vannamei* at different time points (e.g. 1.5h, 18h and 56h). Each cell in the heatmap represents the average expression level from three independent biological replicate samples. Colour legend is on a log10 scale. Trinity contig names matching each gene can be found in Suppl. Table 6.

Supplementary Figure 4: Heatmap of DE genes related with aerobic metabolism. Heatmap showing DE genes involved in maintenance of aerobic metabolism in control (“Ctrl”) and WSSV infected (“WSSV”) *L. vannamei* at different time points (e.g. 1.5h, 18h, 56h). Each cell in the heatmap represents the average expression level from three independent biological replicate samples. Colour legend is on a log10 scale. Trinity contig names matching each gene can be found in Suppl. Table 6.
